# Unmet social needs among low‐income adults in the United States: Associations with health care access and quality

**DOI:** 10.1111/1475-6773.13555

**Published:** 2020-09-03

**Authors:** Megan B. Cole, Kevin H. Nguyen

**Affiliations:** ^1^ Boston University School of Public Health Boston Massachusetts; ^2^ Brown University School of Public Health Providence Rhode Island

**Keywords:** access to care, quality of care, social needs

## Abstract

**Objective:**

To describe social needs among low‐income adults and estimate the relationship between level of unmet social needs and key indicators of health care access and quality.

**Data Source:**

National survey data from 12 states from the 2017 Behavioral Risk Factor Surveillance System, which added a “Social Determinants of Health” Module in 2017.

**Study Design:**

We examined differences in eight measures of health care access and quality (eg, check‐up in last 12 months, inability to see doctor due to cost, receipt of eye examination for diabetics) for low‐income adults with 0, 1, 2‐3, and 4+ unmet social needs based on 7 social needs measures. We used adjusted logistic regression models to estimate the association between level of unmet need and each outcome.

**Principal Findings:**

Most common unmet social needs included not having enough money for balanced meals (33 percent) or food (32 percent). After adjusting for observable characteristics, higher levels of unmet social need were associated with poorer access and quality. Compared to those with no reported unmet needs, having 4+ unmet needs was significantly associated with reduced probability of having a yearly check‐up (65 percent vs 78 percent, adjusted difference = −7.1 percentage points (PP)), receiving a flu vaccine (33 percent vs 42 percent, adjusted difference = −5.4 PP), having a personal doctor (74 percent vs 80 percent, adjusted difference = −3.1 PP), and having a foot (63 percent vs 80 percent, adjusted difference = −12.8 PP) or eye examination (61 percent vs 73 percent, adjusted difference = −12.6 PP) for diabetic patients, and increased probability of being unable to see a doctor due to cost (44 percent vs 9 percent, adjusted difference = 27.9 PP) and having diabetes affect the eyes (22 percent vs 19 percent, adjusted difference = 8.0 PP) at *α* = 0.05.

**Conclusions:**

Higher levels of unmet social needs were associated with poorer access to and quality of care among low‐income adults. Addressing social needs both inside and outside of health care settings may help mitigate these negative effects. Additional research on if and how to effectively do so is critical.

**What This Study Adds**

- Over the last two decades, the critical link between unmet social needs and adverse health outcomes has become increasingly clear, but evidence has been limited to single types of needs and has focused on small, narrowly defined populations.
- In 2017, for the first time, the national Behavioral Risk Factor Surveillance System (BRFSS) survey included an optional “Social Determinants of Health” module for states—producing the first of its kind multi‐state, representative survey data that capture information on multidimensional social needs and health care use.
- This study shows that in a large, multi‐state population of low‐income adults, persons with higher levels of unmet social needs had poorer access to and quality of care compared to persons without unmet needs, after adjusting for sociodemographic and clinical characteristics.
- Findings suggest that addressing unmet social needs across different types of health care and nonhealth care settings may help mitigate the negative association between unmet social needs and access and quality, although additional research on how to effectively do so is critical.

## 1 INTRODUCTION

Over the last two decades, the critical link between unmet social needs, such as food insecurity, housing instability, and financial stress, and adverse health outcomes has become increasingly clear. This is particularly true in low‐income populations, where a recent survey estimates that over 90 percent of persons below 138 percent of the federal poverty level (FPL) have some unmet social needs.^1^ For instance, there is evidence that patients with unmet social needs have higher rates of chronic conditions such as depression and diabetes, are twice as likely to frequently use the emergency department for care, and are more likely to miss scheduled office visits.^2^ This has important implications for the delivery and coordination of care for providers serving populations at high risk of having unmet social needs; underlying this is both a professional imperative for the health care system to maximize health for all patients as well as a financial imperative for providers to do so under shifts to value‐based payment models.

In response, providers and health systems are beginning to consider how to best understand and address the social needs of patients. This includes the implementation of screening protocols that aim to identify unmet needs and link patients to appropriate resources as well as efforts to coordinate nonmedical services that aim to address the social needs of patients.^3^, ^4^, ^5^, ^6^, ^7^ Although, even for health care organizations committed to addressing social needs, many challenges remain in doing so.^8^

While the effect of unmet social needs on quality and health has been documented in the literature,^9^, ^10^, ^11^, ^12^, ^13^ literature to date has been limited to assessing single types of needs (eg, food security), has focused on small, narrowly defined populations (eg, a few primary care practices or cohorts of diabetic patients), and has been largely descriptive in nature. There is no known empirical evidence that assesses unmet social needs within a representative sample of low‐income US adults nor the relationship between unmet social needs and key indicators of access and quality. In order to best identify and reach patients with unmet needs, it is important to first understand characteristics of patients and their types of unmet needs across larger, more representative populations. It is also critical that we understand how these unmet needs may affect access to care and quality of care, as this may provide an impetus for health systems, providers, health plans, or states that are otherwise accountable for quality outcomes to address the social needs of their patients. It may be especially important to understand this association in patients with chronic conditions such as diabetes, for whom the health consequences of unmet needs may be substantial.^14^, ^15^, ^16^

In 2017, for the first time, the national Behavioral Risk Factor Surveillance System (BRFSS) survey included an optional “Social Determinants of Health” module for states—producing the first of its kind multi‐state, representative survey data that capture multidimensional information on both social needs and health care use. As such, our objectives were twofold: (a) to describe the types and extent of unmet social needs among low‐income US adults and (b) to estimate the association between levels of unmet need and key indicators of health care access and quality among low‐income US adults. We hypothesized that those with higher levels of unmet need would have lower rates of access and quality, after adjusting for observable confounding characteristics. We focus on a low‐income population, as this minimizes the confounding role of income on access to and quality of care and allows us to better understand need within a higher‐risk target population.

## 2 METHODS

### 2.1 Data source and study population

We used the 2017 Behavioral Risk Factor Surveillance System (BRFSS) data, which added an optional “Social Determinants of Health” (SDOH) Module in 2017; while termed a “SDOH” Module by BRFSS, the module largely captures information on social needs. BRFSS is a nationally representative, annual health‐related telephone survey that collects state data from US residents regarding health behaviors, chronic conditions, use of preventive services, and access to care.^17^ In 2017, 12 states (Florida, Georgia, Iowa, Massachusetts, Minnesota, Mississippi, New Hampshire, Pennsylvania, Utah, West Virginia, Wisconsin, and Wyoming) collected data for the “SDOH” Module. The survey uses a random digit dial technique with both landline and cellular phone numbers, where the response rate in 2017 across all states was 45.3 percent for landlines and 44.5 percent for cell phones.^18^

We limited our study sample to low‐income (<=200 percent of the FPL) adult (age ≥ 18) respondents from the 12 states reporting the “SDOH” Module. We identified those at or under 200 percent FPL based on reported income and household size, using the 2017 Department of Health and Human Services poverty guidelines.^19^ After excluding respondents who skipped the “SDOH” Module (n = 3360), our final primary study sample included 19 454 respondents, representing a population of 10.95 million low‐income adults. Our secondary study sample included 2128 respondents with diabetes, representing 1.16 million low‐income diabetic adults.

### 2.2 Exposure definition

Our exposure of interest was categorical level of unmet social need or social vulnerabilities (categorized as 0, 1, 2‐3, or 4+), based on seven dichotomized “SDOH” survey questions, referred to herein as unmet social needs. The seven survey questions included the following: (1) Are you not able to pay your mortgage, rent, or utility bills?, (2) Do you not have enough money to make ends meet at end of month?, (3) Do you have no money for food?, (4) Do you have no money for balanced meals?, (5) Did you move more than once during the year?, (6) Do you feel stress most or all of the time?, and (7) Do you consider your neighborhood to be unsafe or extremely unsafe? The original survey questions are listed in Appendix S1.

In our primary analysis, when treating unmet needs as a categorical variable, 8897 respondents (46 percent) reported no unmet needs, 3500 (18 percent) reported one unmet need, 4456 (23 percent) reported 2‐3 unmet needs, and 2601 (13 percent) reported 4 or more unmet needs, out of a maximum score of seven.

### 2.3 Key outcome measures

We examined eight key measures of access and quality of care that we thought may be associated with unmet social needs and that were reported in all or most of the 12 study states. Our four primary outcome measures included having a check‐up in the last 12 months, receiving a flu vaccine in the last 12 months, having a personal doctor or health care provider, and inability to see a doctor because of cost. Among diabetic patients only, we examined four secondary quality outcomes: receiving two or more diabetes tests in the last 12 months, receiving at least one foot examination in the last 12 months, receiving an eye examination in the last 12 months, and whether diabetes was affecting the patient's eyes or resulting in retinopathy.

### 2.4 Statistical analysis

First, we generated survey‐weighted summary statistics to describe rates of unmet social needs for each of the seven survey items. Second, we used chi‐square tests to compare important sociodemographic and clinical characteristics across patients with different levels of unmet social needs.

Next, we estimated the association between level of unmet social needs and each of our study outcomes. To do so, we used logistic regression models to estimate the relationship between level of social needs and each of our binary outcome measures. All models adjusted for age, sex, race/ethnicity, insurance status, educational attainment, income‐level, self‐rated health status, and indicators for high blood pressure, current smoking status, heavy drinking, poor mental health status, asthma, diabetes, and depression, used state fixed effects, accounted for the complex survey weights in the dataset, and used Taylor linearized variance estimation; details on how each covariate was measured are included in Appendix S1. For each outcome, we calculated average marginal effects for each level of social need, reported as the absolute difference in the probability of the outcome as compared to the reference group (respondents with no reported unmet needs).

### 2.5 Sensitivity analyses

We performed multiple sensitivity analyses to assess whether our results were robust under alternative inclusion criteria and methodological specifications. First, we ran our primary models as unadjusted models. Second, we ran all models using stabilized, truncated inverse probability of treatment weights (IPTWs) as to calculate average treatment effects,^20^, ^21^, ^22^, ^23^ based on propensity scores generated from a multiple logistic regression model, where the covariates that were included in the regression adjustment of the primary analysis were included in the propensity score. The use of propensity scores served to balance on observable characteristics between those with higher vs lower levels of unmet social need while reducing the issue of overfitting under the direct adjustment approach.^24^ Third, rather than limiting our sample to low‐income adults only, we re‐ran our primary analyses to assess effects across adults of all income levels; these results are more generalizable to all US adults, but also introduce a greater degree of confounding due to unobservable factors across income strata, as income is highly associated with both unmet social needs and access to and quality of care. Finally, we examined the relationship between each of the seven individual social needs measures and each of our four primary outcome measures to better understand which social needs were most strongly associated with quality and access.

## 3 RESULTS

### 3.1 Characteristics of low‐income adults by level of unmet social need

Prior to adjusting for patient demographics and clinical covariates, there were important differences among low‐income adults across levels of unmet social needs (Table 1). Those with higher levels of unmet need were more likely to be under age 65, Black, uninsured, under 100 percent FPL, current smokers, and identify as lesbian, gay, bisexual, transgender, or other. Those with 4+ unmet needs were 2.4 times as likely to report being in fair or poor health, were 8.2 times as likely to report poor mental health, were 3.1 times as likely to have asthma, and were 4.1 times as likely to have depression, as compared to low‐income adults without any reported unmet needs.

**TABLE 1:** Unadjusted characteristics of low‐income adults by level of unmet social needs (2017)

	Number of unmet social needs				*χ* ^2^
	0 N = 9711	1 N = 3282	2‐3 N = 4023	4+ N = 2438	
Age category					
18‐24	10%	13%	14%	10%	
25‐34	14%	18%	20%	24%	
35‐44	13%	16%	20%	22%	
45‐54	11%	14%	15%	20%	
55‐64	15%	14%	16%	17%	
65+	37%	24%	15%	7%	<0.001
Male	42%	43%	39%	32%	<0.001
Race/ethnicity					
Non‐Hispanic White	67%	57%	56%	58%	
Non‐Hispanic Black	15%	18%	22%	23%	
Non‐Hispanic other race	4%	5%	4%	3%	
Non‐Hispanic multi‐race	1%	2%	2%	3%	
Hispanic	13%	18%	17%	13%	<0.001
Income‐level					
0%‐100% FPL	18%	32%	42%	51%	
101%‐200% FPL	82%	68%	58%	49%	<0.001
Insured	88%	82%	79%	79%	<0.001
Some college or higher	40%	39%	37%	38%	0.033
LGBT+	5%	7%	7%	10%	<0.001
Current smoker	16%	21%	30%	45%	<0.001
Heavy drinker	4%	5%	4%	5%	0.154
Fair or poor self‐rated health	21%	29%	37%	50%	<0.001
Poor self‐rated mental health	6%	15%	25%	47%	<0.001
Asthma	8%	9%	15%	25%	<0.001
Diabetes	14%	16%	16%	16%	<0.001
High blood pressure	40%	40%	37%	41%	0.096
Depression	14%	22%	34%	57%	<0.001
Live in Medicaid expansion state	47%	44%	46%	44%	0.009

Note

N = 19 454 respondents, representing a population of 10.95 million low‐income adults. + LGBT + indicates a sexual orientation or gender identity of lesbian, gay, bisexual, transgender, other, or unsure. Sexual orientation and gender identity were only available for 8 of the 12 reporting states, and thus, statistics may not be representative of the study sample. FPL is federal poverty level. *χ* ^2^ column indicates the p‐value for the chi‐square test for differences in proportions. All estimates are survey weighted.

### 3.2 Types of unmet social needs among low‐income adults

**FIGURE 1:**
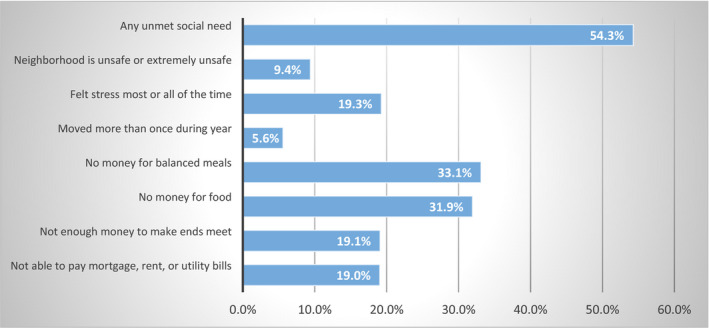
Types of unmet social needs among low‐income adults (2017). [Colour figure can be viewed at wileyonlinelibrary.com] Notes: Any unmet social need indicates a positive need on any one of the 7 reported social needs. All estimates are survey weighted. Source: Author calculations from the 2017 Behavioral Risk Factor Surveillance System.

As shown in Figure 1, across our study sample of low‐income adults, over half (54 percent) of all respondents reported at least one unmet social need out of the seven that were identified. Food insecurity was the most prevalent unmet need, with 32 percent of adults reporting not having enough money for food and 33 percent reporting not having enough money for balanced meals. Financial insecurity—as indicated by not having enough money to make ends meet or pay the bills—was indicated by 19 percent of adults. About 19 percent of low‐income adults reported feeling stress most or all of the time, 9 percent felt that their neighborhoods were unsafe or extremely unsafe, and 6 percent had moved more than once during the year.

### 3.3 Unmet social needs and access to and quality of care

Compared to respondents with no reported unmet social needs, having 2‐3 or 4 or more unmet social needs was associated with statistically lower levels of access and quality for all four of the primary outcomes that were examined (Table 2), after adjusting for observable patient characteristics. For instance, low‐income adults with four or more unmet social needs had a lower probability of having a check‐up in the last 12 months (adjusted difference = −7.1 percentage points (PP), 95% confidence interval (CI): −9.3 to −5.0) relative to those with no reported unmet needs, with unadjusted rates of 65 percent vs 78 percent reporting a check‐up, respectively. Those with four or more unmet social needs also had lower rates of receiving a flu shot in the last 12 months (33 percent vs 42 percent, adjusted difference = −5.4 PP, 95% CI: −7.7 to −3.1) and having a personal doctor (74 percent vs 80 percent, adjusted difference=−3.1 PP, 95% CI: −4.8 to −1.4), while their inability to see a doctor due to cost was much greater (44 percent vs 9 percent, adjusted difference = 27.9 PP, 95% CI: 23.7 to 32.1), as compared to patients with no reported unmet needs. For all four of these measures, as the number of unmet social needs increased, the magnitude of effect increased.

**TABLE 2:** Association between Level of Unmet Social Need and Health Care Quality and Access in Low‐Income Adults (2017)

	Unadjusted outcomes by number of unmet social needs				Adjusted Regression Results			
	0	1	2‐3	4+	Difference in probability	*P*‐value	95% confidence interval	
Check‐up in last 12 mo	78.0%	73.2%	71.3%	65.4%				
0					*ref*	*ref*	*ref*	*ref*
1					−1.4	.094	−2.9	0.2
2‐3					−3.7	<.001	−5.3	−2.2
4+					−7.1	<.001	−9.3	−5.0
Flu shot in last 12 mo	41.7%	35.5%	35.1%	32.7%				
0					*ref*	*ref*	*ref*	*ref*
1					−1.5	.113	−3.4	0.4
2‐3					−2.3	.012	−4.2	−0.5
4+					−5.4	<.001	−7.7	−3.1
Have a personal doctor	80.2%	77.0%	74.3%	74.0%				
0					*ref*	*ref*	*ref*	*ref*
1					−0.7	.311	−2.0	0.7
2‐3					−2.2	.001	−3.5	−0.8
4+					−3.1	<.001	−4.8	−1.4
Inability to see doctor due to cost	9.1%	17.7%	29.8%	43.9%				
0					*ref*	*ref*	*ref*	*ref*
1					7.0	<.001	4.1	9.8
2‐3					17.0	<.001	14.1	19.9
4+					27.9	<.001	23.7	32.1

Note

N = 19 454 respondents, representing a population of 10.95 million low‐income adults. Adjusted outcomes represent mean marginal effects from our adjusted regression models and are reported as absolute percentage point differences. All regression estimates adjust for age, sex, race/ethnicity, insurance status, self‐rated health status, educational attainment, income‐level, state, and indications for high blood pressure, current smoking status, heavy drinking, poor mental health status, asthma, diabetes, and depression. A difference in probability that is <0 means that the outcome was lesser for those with higher levels of unmet need, as compared to those without reported unmet needs (reference group).

In our secondary analyses among low‐income adults with diabetes (Table 3), those with four or more unmet social needs were less likely to receive a foot examination in the last year (unadjusted rate of 63 percent vs 80 percent, adjusted difference = −12.8 PP, 95% CI: −24.1 to −1.5), were less likely to receive an eye examination in the last year (61 percent vs 73 percent, adjusted difference = −12.6 PP, 95% CI: −24.7 to −0.5), and were more likely to have had their diabetes affect their eyes (22 percent vs 19 percent, adjusted difference = 8.0 PP, 95% CI: 1.5 to 14.4), compared to those with no reported unmet social needs. Having at least two glucose tests was not statistically associated with level of unmet need.

**TABLE 3:** Association between level of unmet social need and health care quality in Low‐Income Adults with Diabetes (2017)

	Unadjusted outcomes by number of unmet social needs				Adjusted Regression Results			
	0	1	2‐3	4+	Difference in probability	*P*‐value	95% confidence interval	
2+ glucose tests in last 12 mo	77.6%	75.6%	75.8%	77.3%				
0					*ref*	*ref*	*ref*	*ref*
1					−0.8	.869	−10.7	9.1
2‐3					−0.2	.963	−0.84	8.0
4+					0.6	.915	−10.5	11.7
Foot examination in last 12 months	80.4%	79.2%	77.0%	63.4%				
0					*ref*	*ref*	*ref*	*ref*
1					0.0	.988	−8.3	8.3
2‐3					−0.7	.837	−8.4	6.8
4+					−12.8	.027	−24.1	−1.5
Eye examination in last 12 months	73.1%	65.5%	68.7%	61.2%				
0					*ref*	*ref*	*ref*	*ref*
1					−7.8	.148	−18.6	2.8
2‐3					−4.1	.381	−13.4	5.1
4+					−12.6	.041	−24.7	−0.5
Diabetes has affected eyes	18.7%	26.4%	23.6%	21.9%				
0					*ref*	*ref*	*ref*	*ref*
1					5.2	.040	0.3	10.2
2‐3					4.2	.069	−0.3	8.6
4+					8.0	.016	1.5	14.4

Note

N = 2128 respondents with diabetes, representing 1.16 million low‐income diabetic adults. Adjusted outcomes represent mean marginal effects from our adjusted regression models and are reported as absolute percentage point differences. All regression estimates adjust for age, sex, race/ethnicity, insurance status, self‐rated health status, educational attainment, income‐level, state, and indications for high blood pressure, current smoking status, heavy drinking, poor mental health status, asthma, and depression. A difference in probability that is <0 means that the outcome was lesser for those with higher levels of unmet need, as compared to those without reported unmet needs (reference group).

### 3.4 Subgroup and sensitivity analyses

Results from our sensitivity analyses were largely consistent with our main findings (Appendix S1). Not adjusting for patient characteristics or clinical covariates most often resulted in larger effect estimates of the relationship between unmet need and each of our study outcomes. When applying IPTWs based on propensity scores to our models, results were qualitatively similar but generally had a higher degree of statistical significance, as standard errors were smaller. When assessing the relationship between each individual social need measure and our four primary outcome measures, inability to see a doctor due to cost was most strongly and consistently associated with each of the seven unmet social needs. Finally, when rerunning our primary analyses using adults of all income levels, results were qualitatively similar to our main findings; however, due to larger sample sizes coupled with the confounding role of income, somewhat larger and more statistically significant effects were observed. Full sensitivity results are shown in Appendix S1.

## 4 DISCUSSION

Our study describes unmet social needs across a large, multi‐state, representative population of low‐income adults and examines the relationship between level of unmet social needs and key indicators of access and quality. We find that over half of all low‐income adults reported at least one unmet need, with food insecurity being the most common type of unmet need. Those with higher levels of unmet need were much more likely to be under 65, Black, uninsured, under 100 percent FPL, current smokers, from a sexual‐ or gender‐minority group, and in fair or poor health, and had higher rates of poor mental health, asthma, and depression. Compared to low‐income adults without any reported unmet needs, those with four or more unmet needs were significantly less likely to have a check‐up, receive a flu shot, and have a personal doctor; were much more likely to be unable to see a doctor due to cost; and among diabetic patients, were less likely to receive a yearly foot examination or eye examination and more likely to have diabetes complications affect the eyes.

Even after adjusting for important determinants of access and quality such as insurance coverage and physical and mental health status, having higher levels of unmet social needs or other social vulnerabilities impeded on access to and quality of care for low‐income adults. Here, a patient under financial stress and without sufficient social supports may have significant competing priorities that make it especially challenging to make it to a check‐up; for diabetic patients in particular, this foregone care could have significant health consequences, such as diabetic retinopathy due to poorly controlled blood sugar, which may be further compounded by the patient's inability to purchase food or balanced meals.^25^ To the extent that such patients’ social needs can be appropriately identified and addressed, improvements in quality and access may result.

Higher levels of unmet social needs among low‐income adults appeared to be most strongly associated with being unable to see a doctor due to cost, even after adjusting for insurance status. For respondents experiencing financial insecurities or making decisions about whether to pay for health care or food, this likely serves as a barrier to accessing necessary health care services, particularly services that may be subject to cost sharing in the form of a deductible or copayment. This is especially concerning given that patients with higher levels of unmet social needs also had higher levels of physical and mental health needs, yet had the least access. While information on plan type and benefit structure was unavailable in the data, ensuring that low‐income patients with unmet social needs have access to health insurance with minimal cost sharing may be important to minimizing this access barrier.

This work adds to the growing evidence base on the relationship between unmet social needs and health care quality, although it is the first known study to use large, population‐level data across multiple states and is the only known study to look across multiple dimensions of social need as related to quality and access in the United States. For instance, a 2015 study of two urban academic practices found that difficulty affording or receiving health care (47 percent) and difficulty affording food (40 percent) were the most common types of needs among those with at least one identified need. Unadjusted results from the study suggested that having any unmet need was associated with a greater likelihood of depression, hypertension, diabetes, ED use, missing a clinic appointment, high LDL cholesterol, and .high blood pressure levels.^2^ Recent regional research in Canada has further shown that social complexity factors are associated with reduced quality of primary care.^26^ Many other recent studies have described the relationship between single types of unmet needs (eg, food insecurity) for specific patient populations (eg, diabetic patients, hypertensive patients) and have found unmet needs to be associated with poorer health outcomes.^9^, ^10^, ^11^, ^12^, ^13^, ^14^, ^15^, ^16^, ^27^ We add to this literature by focusing on a more generalizable study population spanning 12 states and by using a survey instrument that was administered outside of a health care setting.

Our findings have five important implications. First, given the high rates of reported unmet social needs within this study population, our results echo the potential importance of screening for and/or addressing social needs, especially those related to food insecurity, financial insecurity, and stress, which we found to be the most pervasive of those included in our study. Despite the fact that up to 90 percent of health outcomes are explained by factors outside health care,^28^ a third of physician practices do not screen for any social needs while 70 percent do not screen for food security,^29^ although the effectiveness of screening for and addressing social needs in medical care settings still remains unclear and understudied in the literature.^30^, ^31^, ^32^, ^33^ Importantly, both our health care and social services systems should consider not only how to best identify needs during regularly scheduled care, but given that those with the highest levels of need are less likely to have an annual check‐up or have a personal doctor, screening and addressing needs outside of health care settings, such as in criminal justice systems, or in emergency departments, where patients with depression or asthma, or without insurance, may be more likely to present, may be especially important. Doing so may entail not only implementing screening protocols across different touch points in the system, but establishing the appropriate staff, workflows, resources, and partnerships that appropriately link patients with social services, while ensuring that those patients receive all necessary health care services. As there is very little evidence to date on if or how to effectively do so,^30^, ^31^, ^32^ and organizations continue to face substantial challenges in trying to screen for and address social needs,^8^ additional research on how to effectively screen for and address social needs across systems is critical.

Second, the high prevalence of unmet needs coupled with its negative association with quality of care may provide an additional impetus for health systems and health plans that are not yet addressing social needs, particularly in an era of value‐based payment. Unmet needs may constrain a system's ability to achieve quality benchmarks, as both preventive and chronic disease management performance measures suffer if patients do not come in for a check‐up or are otherwise unable to see a doctor. Diabetes performance measures are also included in CMS’ Core Measures set^34^ and are part of most ACO and patient‐centered medical home contracts,^35^, ^36^, ^37^ where failing to meet diabetes care benchmarks may result in organizations forgoing shared savings or incentive payments. Addressing the social needs of these patients within the ACO or medical home may be one way to mitigate poor quality outcomes and access.

Third, to the extent that cost remains a barrier to accessing quality health care for low‐income patients with unmet social needs, even when accounting for insurance status, there exist opportunities to mitigate this barrier outside of a clinical setting. Health plans may wish to screen for social needs at time of enrollment and establish supports to ensure that patients with higher levels of need have adequate access to care as well as linkages to social supports as needed, while minimizing cost‐related barriers to care for these persons. Employers providing health insurance to lower‐income employees should also consider their role in addressing unmet social needs, and may consider directly subsidizing cost sharing for their lower‐income employees, particularly those with identified unmet social needs. Lastly, as persons under 100 percent FPL in states without expanded Medicaid eligibility have no access to cost‐sharing subsidies via the Marketplace, this may compound the effects of unmet social needs on access and quality. Expanding Medicaid eligibility to at least 138 percent FPL in all states would enable better access to both health care and to the social supports that may be integrated into health care.

Fourth, as both health care and social service organizations strive to optimize their abilities to adequately address social needs, prioritizing needs such as food security—which affects about one in three low‐income persons in our study yet is only screened for by about 30 percent of physician offices^29^—could help to mitigate the negative association with quality and health care use. For example, starting in 2020, the Massachusetts Medicaid Accountable Care Organization (ACO) model is allocating $149 million for ACOs to spend on flexible services, where ACOs may use allocated flexible service dollars to address social needs of their qualifying patients who are food insecure or housing insecure.^38^ Addressing food security through a program such as home‐delivered meals, for example, may not only combat food insecurity but could also potentially improve access and quality outcomes for patients, as supported in other literature.^39^, ^40^

Finally, more broadly, structural and policy‐level efforts should be made to address the root causes of unmet social needs, particularly for those experiencing disproportionate levels of need such as Black, uninsured, and sexual‐ and gender‐minority persons, as identified in our study. Addressing such inequities will require cross‐sectoral collaboration and policy changes that aim to improve the social and economic conditions of marginalized communities. Efforts to systematically dismantle structural discrimination across sectors, expand health insurance coverage, and create more targeted resources and social supports for marginalized groups, for instance, could mitigate inequities in unmet needs, thereby leading to improvements in access and quality of care.

Our study has several important limitations. First, because the BRFSS “SDOH” Module was fielded in a single year (2017) only, we are unable to assess changes over time or estimate effects of specific policies. However, this represents the only known current multi‐state data to assess both multidimensional social needs and health care use. Second, the composition of our unmet social needs score is limited by what is collected in the survey data; no questions measure needs pertaining to transportation or intimate partner violence, for instance, and thus, we are unable to capture those dimensions of social needs. While the survey does not explicitly ask about homelessness or housing security, we are able to capture housing stability by using number of times moved in a year as a proxy measure. Third, we are further limited by quality and access outcomes that are reported by all 12 states in our study sample. For instance, optional questions regarding difficultly accessing medications due to cost and satisfaction with care were only available for a small number of states, and thus, we are unable to evaluate these outcomes. Fourth, because we focus on a low‐income population, our primary effect estimates are more conservative than those in the general population. However, limiting our population to low‐income persons minimizes the confounding role of income while focusing on a population at higher risk of both unmet social needs and poorer health care quality and access. Fifth, our models adjust for health‐related covariates such as depression and self‐rated mental health, which may serve as both confounders and as factors on the causal pathway. By adjusting for these factors, our effect estimates are likely conservative. Sixth, our estimates should further be interpreted as conservative given that those experiencing homelessness or without a reliable telephone are unlikely to participate in the BRFSS, despite likely having high levels of unmet social needs. Finally, our findings represent associations only, though they represent the only known national data on these questions.

## 5 CONCLUSIONS

National survey data suggest that there are substantial unmet social needs across low‐income adults, particularly as related to food security. Unmet social needs were strongly associated with poorer access to and quality of care among lower‐income adults. Identifying and addressing social needs both inside and outside of doctors’ offices may help mitigate the negative association between unmet social needs and access and quality. Developing an evidence base on effective strategies for doing so is critical.

## Supporting information

Supplementary Material

Appendix S1
