# Supplementary material for: Unmet social needs among low‐income adults in the United States: Associations with health care access and quality
Source: Health Serv Res. 2020 Sep 3;55(Suppl 2):873–82. doi: 10.1111/1475-6773.13555 (PMC7518813; doi:10.1111/1475-6773.13555)
Supplement: Supplementary file 2 — Appendix S1 [file HESR-55-873-s002.docx]

**Appendix S1**

**Table of Contents**

[Table S1. List of original survey questions measuring unmet social needs 2](#_Toc36633339)

[Table S2. Covariate definitions 3](#_Toc36633340)

[Table S3a. Sensitivity Analysis: Unadjusted Association between Level of Unmet Social Need and Health Care Quality and Access in Low-Income Adults (2017) 5](#_Toc36633341)

[Table S3b. Sensitivity Analysis: Unadjusted Association between Level of Unmet Social Need and Health Care Quality in Low-Income Adults with Diabetes (2017) 6](#_Toc36633342)

[Table S4. Sensitivity Analysis: Association between Level of Unmet Social Need and Health Care Quality and Access in Low-Income Adults with Inverse Probability of Treatment Weights (2017) 7](#_Toc36633343)

[Table S5. Sensitivity Analysis: Association between Level of Unmet Social Needs and Health Care Quality and Access across Adults of All Income Levels (2017) 8](#_Toc36633344)

[Table S6. Sensitivity Analysis: Association between Unmet Social Need and Health Care Quality and Access in Low-Income Adults by Individual Social Needs Measure (2017) 9](#_Toc36633345)

[Table S7. Covariate Balance for Low-Income Adults by Level of Social Need: Standardized Differences Before vs. After Propensity Weighting 10](#_Toc36633346)

Table S1. List of original survey questions measuring unmet social needs

| **Question** | **Response options (excludes don’t know/refused/missing)** | **Unmet need classification** |
| --- | --- | --- |
| During the last 12 months, was there a time when you were not able to pay your mortgage, rent or utility bills? | 1. Yes 2. No | 1 |
| In the last 12 months, how many times have you moved from one home to another? | 1 – 52: Number of times moved  88: Did not move | 2-52 |
| How safe from crime do you consider your neighborhood to be? | 1. Extremely safe 2. Safe 3. Unsafe 4. Extremely unsafe | 3, 4 |
| “The food that I bought just didn’t last, and I didn’t have money to get more.” Was that  often, sometimes, or never true for you in the last 12 months? | 1. Often true 2. Sometimes true 3. Never true | 1, 2 |
| “I couldn’t afford to eat balanced meals.” Was that often, sometimes, or never true for you in the last 12 months? | 1. Often true 2. Sometimes true 3. Never true | 1, 2 |
| In general, how do your finances usually work out at the end of the month? | 1. End up with some money left over 2. Have just enough money to make ends meet 3. Not have enough money to make ends meet | 3 |
| Stress means a situation in which a person feels tense, restless, nervous, or anxious, or is unable to sleep at night because his/her mind is troubled all the time.  Within the last 30 days, how often have you felt this kind of stress? | 1. None of the time 2. A little of the time 3. Some of the time 4. Most of the time 5. All of the time | 4, 5 |

Table S2. Covariate definitions

| **Covariate** | **Operational definition** |
| --- | --- |
| Age | Age category based on the following categories:   - 18-24 - 25-34 - 35-44 - 45-54 - 55-64 - 65+ |
| Sex | male, female |
| Race/ethnicity | Racial/ethnicity based on the following mutually exclusive and collectively exhaustive categories:   - Non-Hispanic White - Non-Hispanic Black - Non-Hispanic other race - Non-Hispanic multi-race - Hispanic |
| Income-level | Income calculated as categorical percent of the federal poverty level based on the reported household income and household size (total # of adults and children in household), using the 2017 Department of Health and Human Services poverty guidelines. Categories included:   - 0-100% FPL - 101-200% FPL   More precise calculation of income level not feasible with data given the categorical nature of total household income reporting. |
| Insurance status | Whether or not the respondent had any insurance coverage (yes/no) based on the following question: “Do you have any kind of health care coverage, including health insurance, prepaid plans such as HMOs, or government plans such as Medicare, or Indian Health Service?” |
| Educational attainment | Whether or not the respondent received any college education (yes/no) based on the highest grade/year completed:   - No: Never attended school; Grades 1-8; Grades 9-11; Grade 12 or GED - Yes: College 1 year to 3 years (Some college or technical school) ; College 4 years or more (College graduate) |
| LGBT+ | Whether or not the respondent identified as a sexual or gender minority (yes/no):   - No: straight and not transgender - Yes: sexual orientation or gender identity is lesbian, gay, bisexual, transgender, or other |
| Self-rated health | Whether or not the respondent identified as having fair or poor health (yes/no) based on the following question: Would you say that in general your health is:   - No: excellent, very good, good - Yes: fair, poor |
| Self-rated mental health | Whether or not the respondent reported poor mental health (yes/no) based on the number of days in the month during which mental health was not good:   - No: 0 days, 1-13 days - Yes: 14+ days |
| Medicaid expansion state | Whether or not the respondent lived in a Medicaid expansion state (yes/no) as of 2017:   - No: FL, GA, MS, UT, WI, WY - Yes: IA, MA, MN, NH, PA, WV |
| Indication of high blood pressure, diabetes, asthma, or depression | Whether or not the respondent was ever told (yes/no) that they had the respective condition. |
| Current smoking status | Whether or not the respondent indicated that they currently smoke cigarettes (yes/no):   - No: former smoker, never smoker - Yes: Current smoker - now smokes every day; Current smoker - now smokes some days |
| Heavy drinker | Whether or not the respondent was calculated as having heavy alcohol consumption (yes/no):   - No: <=14 drinks/week for males; <=7 drinks/week for females; or zero drinks in the past 30 days - Yes: >14 drinks/week for males; >7 drinks/week for females |

Table S3a. Sensitivity Analysis: Unadjusted Association between Level of Unmet Social Need and Health Care Quality and Access in Low-Income Adults (2017)

|  | Unadjusted outcomes by number of unmet social needs | | | | Unadjusted Regression Results | | | |
| --- | --- | --- | --- | --- | --- | --- | --- | --- |
|  | 0 | 1 | 2-3 | 4+ | Difference in probability | p-value | 95% | CI |
| Check-up in last 12 months | 78.0% | 73.2% | 71.3% | 65.4% |  |  |  |  |
| 0 |  |  |  |  | *ref* | *ref* | *ref* | *ref* |
| 1 |  |  |  |  | -4.8 | 0.007 | -8.2 | -1.3 |
| 2-3 |  |  |  |  | -6.7 | <0.001 | -9.8 | -3.7 |
| 4+ |  |  |  |  | -12.6 | <0.001 | -16.3 | -8.9 |
| Flu shot in last 12 months | 41.7% | 35.5% | 35.1% | 32.7% |  |  |  |  |
| 0 |  |  |  |  | *ref* | *ref* | *ref* | *ref* |
| 1 |  |  |  |  | -6.2 | 0.001 | -9.9 | -2.4 |
| 2-3 |  |  |  |  | -6.6 | <0.001 | -10.0 | -3.1 |
| 4+ |  |  |  |  | -9.0 | <0.001 | -12.9 | -5.0 |
| Have a personal doctor | 80.2% | 77.0% | 74.3% | 74.0% |  |  |  |  |
| 0 |  |  |  |  | *ref* | *ref* | *ref* | *ref* |
| 1 |  |  |  |  | -3.2 | 0.074 | -6.6 | 0.3 |
| 2-3 |  |  |  |  | -5.9 | <0.001 | -9.0 | -2.8 |
| 4+ |  |  |  |  | -6.2 | 0.002 | -10.0 | -2.3 |
| Inability to see doctor due to cost | 9.1% | 17.7% | 29.8% | 43.9% |  |  |  |  |
| 0 |  |  |  |  | *ref* | *ref* | *ref* | *ref* |
| 1 |  |  |  |  | 8.6 | <0.001 | 5.8 | 11.4 |
| 2-3 |  |  |  |  | 20.7 | <0.001 | 17.7 | 23.7 |
| 4+ |  |  |  |  | 34.8 | <0.001 | 31.0 | 38.6 |

*Source:* Author calculations from the 2017 Behavioral Risk Factor Surveillance System

*Notes:* N=19,454 respondents, representing a population of 10.95 million low-income adults. Unadjusted outcomes are derived from logistic regression models and represent mean marginal effects. A difference in probability that is <0 means that the outcome was lesser for those with higher levels of unmet need, as compared to those without reported unmet needs (reference group).

Table S3b. Sensitivity Analysis: Unadjusted Association between Level of Unmet Social Need and Health Care Quality in Low-Income Adults with Diabetes (2017)

|  | Unadjusted outcomes by number of unmet social needs | | | | Unadjusted Regression Results | | | |
| --- | --- | --- | --- | --- | --- | --- | --- | --- |
|  | 0 | 1 | 2-3 | 4+ | Difference in probability | p-value | 95% | CI |
| 2+ glucose tests in last 12 months | 77.6% | 75.6% | 75.8% | 77.3% |  |  |  |  |
| 0 |  |  |  |  | *ref* | *ref* | *ref* | *ref* |
| 1 |  |  |  |  | -2.0 | 0.682 | -11.8 | 7.7 |
| 2-3 |  |  |  |  | -1.8 | 0.668 | -9.9 | 6.3 |
| 4+ |  |  |  |  | -0.4 | 0.946 | -10.6 | 9.9 |
| Foot exam in the last 12 months | 80.4% | 79.2% | 77.0% | 63.4% |  |  |  |  |
| 0 |  |  |  |  | *ref* | *ref* | *ref* | *ref* |
| 1 |  |  |  |  | -1.2 | 0.787 | -10.0 | 7.6 |
| 2-3 |  |  |  |  | -3.4 | 0.405 | -11.6 | 4.7 |
| 4+ |  |  |  |  | -17.1 | 0.010 | -30.1 | -4.1 |
| Eye exam in last 12 months | 73.1% | 65.5% | 68.7% | 61.2% |  |  |  |  |
| 0 |  |  |  |  | *ref* | *ref* | *ref* | *ref* |
| 1 |  |  |  |  | -7.6 | 0.144 | -17.7 | 2.6 |
| 2-3 |  |  |  |  | -4.4 | 0.347 | -13.5 | 4.7 |
| 4+ |  |  |  |  | -11.9 | 0.050 | -23.8 | 0.0 |
| Diabetes has affected eyes | 18.7% | 26.4% | 23.6% | 21.9% |  |  |  |  |
| 0 |  |  |  |  | *ref* | *ref* | *ref* | *ref* |
| 1 |  |  |  |  | 5.9 | 0.022 | 0.9 | 10.9 |
| 2-3 |  |  |  |  | 4.7 | 0.036 | 0.3 | 9.1 |
| 4+ |  |  |  |  | 8.8 | 0.002 | 3.1 | 14.5 |

*Source:* Author calculations from the 2017 Behavioral Risk Factor Surveillance System

*Notes:* N= 2,128 respondents with diabetes, representing 1.16 million low-income diabetic adults. Unadjusted outcomes are derived from logistic regression models and represent mean marginal effects. A difference in probability that is <0 means that the outcome was lesser for those with higher levels of unmet need, as compared to those without reported unmet needs (reference group).

Table S4. Sensitivity Analysis: Association between Level of Unmet Social Need and Health Care Quality and Access in Low-Income Adults with Inverse Probability of Treatment Weights (2017)

*Source:* Author calculations from the 2017 Behavioral Risk Factor Surveillance System

*Notes:* N=19,454 respondents, representing a population of 10.95 million low-income adults. Adjusted outcomes are derived from logistic regression models and represent mean marginal effects. IPTW are inverse probability of treatment weights, based on propensity scores, which balance on age, sex, race/ethnicity, insurance status, self-rated health status, educational attainment, income-level, state Medicaid expansion status, survey weights, and indications for high blood pressure, current smoking status, heavy drinking, poor mental health status, asthma, diabetes, and depression across levels of unmet need. A coeff <0 means that the outcome was lesser for those with higher levels of unmet need, as compared to those without reported unmet needs (reference group).

Table S5. Sensitivity Analysis: Association between Level of Unmet Social Needs and Health Care Quality and Access across Adults of All Income Levels (2017)

*Source:* Author calculations from the 2017 Behavioral Risk Factor Surveillance System

*Notes:* N= 72,826 respondents, representing 39.8 million adults across all income levels. Adjusted outcomes represent marginal effects from our adjusted regression models. All regression estimates adjust for age, sex, race/ethnicity, insurance status, self-rated health status, educational attainment, income-level, state, survey weights, and indications for high blood pressure, current smoking status, heavy drinking, poor mental health status, asthma, diabetes, and depression. A difference in probability that is <0 means that the outcome was lesser for those with higher levels of unmet need, as compared to those without reported unmet needs (reference group).

Table S6. Sensitivity Analysis: Association between Unmet Social Need and Health Care Quality and Access in Low-Income Adults by Individual Social Needs Measure (2017)

*Source:* Author calculations from the 2017 Behavioral Risk Factor Surveillance System

*Notes:* Adjusted outcomes represent marginal effects from our adjusted regression models. All regression estimates adjust for age, sex, race/ethnicity, insurance status, self-rated health status, educational attainment, income-level, state, survey weights, and indications for high blood pressure, heavy drinking, poor mental health status, asthma, diabetes, and depression. An Odds Ratio (OR) <1.0 means that the outcome was lesser for those with indicator of the unmet need, as compared to those without a reported unmet need (reference group).

Table S7. Covariate Balance for Low-Income Adults by Level of Social Need: Standardized Differences Before vs. After Propensity Weighting

|  | Standardized differences | |
| --- | --- | --- |
|  | Raw | Weighted |
|  |  |  |
| Unmet needs=1 |  |  |
| age category (ref=18-24) |  |  |
| 25-34 | 0.1519452 | 0.0101598 |
| 35-44 | 0.0950365 | -0.0063599 |
| 45-54 | 0.1341735 | -0.0189564 |
| 55-64 | 0.0732738 | -0.0020782 |
| 65+ | -0.3679294 | 0.0104879 |
|  |  |  |
| race/ethnicity (ref=White) |  |  |
| Black | 0.115562 | -0.0468227 |
| other race | 0.0530451 | -0.0071254 |
| multi-race | 0.0486523 | -0.0014926 |
| Hispanic | 0.0944595 | 0.0032055 |
|  |  |  |
| sex (ref=female) | -0.0097207 | 0.0323569 |
| insured | -0.135102 | 0.0146131 |
| fair or poor health | 0.2308126 | -0.0408546 |
| high blood pressure | -0.0260546 | -0.0256617 |
| current smoker | 0.161989 | -0.0058104 |
| heavy drinker | 0.0119293 | -0.0132964 |
| poor mental health | 0.3420818 | -0.0393357 |
| asthma | 0.0772847 | -0.0372523 |
| diabetes | 0.0248572 | -0.0153664 |
| depression | 0.3217627 | -0.025804 |
| any college | -0.0037096 | 0.0122817 |
| survey weight | 0.0673038 | 0.0016502 |
| Unmet needs=2-3 |  |  |
| age category (ref=18-24) |  |  |
| 25-34 | 0.1571105 | 0.0059442 |
| 35-44 | 0.2069825 | -0.0027219 |
| 45-54 | 0.2039069 | -0.0198783 |
| 55-64 | 0.145362 | -0.0067701 |
| 65+ | -0.5942926 | 0.0136857 |
|  |  |  |
| race/ethnicity (ref=White) |  |  |
| Black | 0.1702002 | -0.0287213 |
| other race | 0.0626355 | 0.0040726 |
| multi-race | 0.0797867 | -0.0076578 |
| Hispanic | 0.1042753 | 0.0074974 |
|  |  |  |
| sex (ref=female) | -0.0868448 | 0.0091115 |
| insured | -0.2216849 | 0.0082423 |
| fair or poor health | 0.3953747 | -0.0331838 |
| high blood pressure | -0.0192044 | -0.0183193 |
| current smoker | 0.3729198 | -0.0097559 |
| heavy drinker | 0.0315941 | -0.020767 |
| poor mental health | 0.5657961 | -0.0402078 |
| asthma | 0.2433232 | -0.032313 |
| diabetes | 0.0958282 | 0.0047055 |
| depression | 0.5343853 | -0.0312707 |
| Any college | -0.0539815 | 0.0083282 |
| survey weight | 0.1044219 | -0.0024052 |
| Unmet needs=4+ |  |  |
| age category (ref=18-24) |  |  |
| 25-34 | 0.1687528 | 0.0130498 |
| 35-44 | 0.2576256 | -0.0033118 |
| 45-54 | 0.3944607 | -0.0000874 |
| 55-64 | 0.1968427 | 0.0052808 |
| 65+ | -0.883361 | 0.0083459 |
|  |  |  |
| race/ethnicity (ref=White) |  |  |
| Black | 0.1761667 | 0.0285571 |
| other race | 0.0745051 | -0.0192417 |
| multi-race | 0.1384489 | -0.0029812 |
| Hispanic | 0.0400752 | 0.0218464 |
|  |  |  |
| sex (ref=female) | -0.1705045 | -0.0015816 |
| insured | -0.2585286 | 0.0252713 |
| fair or poor health | 0.7311346 | -0.0042146 |
| high blood pressure | 0.0346897 | -0.0380181 |
| current smoker | 0.671651 | 0.0441304 |
| heavy drinker | 0.0424239 | -0.0709933 |
| poor mental health | 1.120803 | -0.0207723 |
| asthma | 0.4731423 | -0.020302 |
| diabetes | 0.0768603 | 0.0012565 |
| depression | 1.116401 | 0.0024037 |
| any college | -0.0203985 | 0.008244 |
| survey weight | 0.1165502 | 0.0073669 |
